# Helping address the national research and research capacity needs of Australian chiropractic: introducing the Australian Chiropractic Research Network (ACORN) project

**DOI:** 10.1186/s12998-015-0057-8

**Published:** 2015-04-01

**Authors:** Jon Adams, Amie Steel, Sungwon Chang, David Sibbritt

**Affiliations:** Australian Research Centre in Complementary and Integrative Medicine, Faculty of Health, University of Technology, Sydney, Australia; Office of Research, Endeavour College of Natural Health, Brisbane, Australia

**Keywords:** Chiropractic, Research, Practice based research networks, Research capacity building

## Abstract

Chiropractic is a popular health care choice in Australia and yet major gaps in our empirical understanding of this area of practice remain. Furthermore, while some research excellence exists, a largely uncoordinated approach to research activity and development has in effect led to silos of interest and a lack of strategic ‘big-picture’ planning essential to producing a sustainable research culture and capacity for the profession. This commentary identifies the significance of a number of key features - including a national, coordinated focus, and a rich engagement with the practitioner and patient base amongst others – arguably important to the future development of research and research capacity within Australian chiropractic. The design features and phases of the Australian Chiropractic Research Network (ACORN) project are also outlined. ACORN is one contemporary initiative specifically developed to address chiropractic’s research and research capacity building needs and help grow a broad evidence-base to inform safe, effective patient care.

## Background

Chiropractic is a popular health care choice in Australia as elsewhere constituting a major component of health care utilisation (estimated to be worth over $3 M in out-of-pocket expenses in 2005 [1]) and attracting reports of good patient satisfaction [2-10]. Despite emerging research activity and developments within chiropractic in Australia and overseas [11-13], major gaps in our empirical understanding of this area of health care practice remain. There is much room for the further development of a broad range of methods and disciplinary perspectives to help assess and advance the evidence-base for chiropractic care. Given the prevalence of chiropractic use in Australia, further research development - providing insights of benefit for practitioners, patients and policymakers - is essential to ensuring effective, safe, coordinated chiropractic care and provision [14].

A key challenge that has faced research of chiropractic in Australia to date has been the piecemeal and often largely uncoordinated nature of research activity. While some research excellence and emerging evidence exists, a largely uncoordinated approach has in effect led to silos of interest and a lack of strategic ‘big-picture’ planning essential to producing a sustainable research culture for the profession [15]. Following the lead of other health professional groups it is vital that Australian chiropractic invest substantial effort and resources to facilitate a coordinated approach to producing a sustainable culture of research [11,12,15-19]. A failure to significantly invest in research capacity building runs the very real risk of substantially limiting the prospects of the profession and its research base for the foreseeable future [16-20].

## Discussion

### Key features essential to future development of chiropractic research and research capacity building

In order to directly and successfully address the contemporary research and research capacity building needs of Australian chiropractic and to ensure necessary credibility and strength in design, there is an arguably urgent need for initiatives to build upon a number of interrelated, distinct features. In particular, we would argue, it is essential such initiatives wherever possible: be based upon a critical, non-partisan research approach; adopt and build upon rigorous health and medical research methods and designs; be coordinated, broad and multidisciplinary in research focus; be national in design and scope; be longitudinal and sustainable providing short term, mid-term and long term outcomes as well as helping build a wider incremental program of research; incorporate practitioner engagement and research capacity building wherever possible; reflect and connect with the clinical and practice realities of contemporary chiropractic and wider health care in order to ensure translation and impact of results for practitioners and patient care; and be inclusive and research network building (housing an ability to facilitate, promote and advance research networks where already established and help encourage and grow networks and research activity elsewhere as necessary).

### One new strategy of response: introducing the ACORN project

The Australian Chiropractic Research Network (ACORN) project is one contemporary initiative which has been designed and launched in direct response to these research and research capacity building needs. In the following sections of this paper, the overarching design and phases of the ACORN project will be explained along with a discussion of the specific ways in which the project will address the essential research and research capacity building issues facing Australian chiropractic.

ACORN is the first long-term, national project built upon a Practice-Based Research Network (PBRN) design to examine chiropractic care in Australia. A PBRN design has been previously utilised in a range of health care fields including general practice [21-23], pharmacy [24] and dentistry [25]. Chiropractic PBRN work has developed outside Australia [26] and, while not constituting a PBRN, practice-based research has also previously been undertaken in Australian chiropractic on a regional level [4,27]. PBRN studies act as ‘research laboratories’ for health and medical care [22] drawing upon groups of practitioners/practices (small or large in number) in order to systematically answer questions that arise from clinical practice [28] as well as undertake research of relevance to both grass-roots practitioners and patient care [29]. The notion of a PBRN being sustainable and capacity building has also been highlighted [30,31]. Examples of the type of issues that have previously been investigated via PBRN designs include an examination of a range of information sources practitioners draw upon to inform their clinical decision-making [32] and the comparative effectiveness of different models of health care [33]. A PBRN is extremely well positioned to help address research questions around professional standards of practice and clinical characteristics and behaviours and can provide an opportunity for more rigorous sub-studies to examine the efficacy of particular protocols within particular patient sub-groups of *direct relevance* to frontline practice settings [30].

The ACORN project, funded by the Chiropractors’ Association of Australia, has been independently designed and is independently led and conducted by the Australian Research Centre in Complementary and Integrative Medicine (ARCCIM), Faculty of Health, University of Technology Sydney (UTS). The project will provide extensive data collection and analysis as well as develop essential national infrastructure for future research on chiropractic. The ACORN project draws upon and promotes critical multidisciplinary, longitudinal investigation based upon rigorous scientific design and methods, strongly encourages (and relies upon) collaborative involvement from across the chiropractic profession – both in terms of practitioners and researchers/research groups – and introduces a national coordinated agenda for chiropractic research that is translational in output and reflective of practice realities. While the project is capable of facilitating a diverse range of research sub-studies and addressing a very broad range of distinct but interrelated research questions, the platform focus of the ACORN project is upon examining issues around chiropractic best practice, safety and cost-effectiveness. The ACORN project will employ a diverse dissemination strategy to ensure empirical findings reach practitioner, patient, researcher and policymaker audiences. Dissemination of the empirical findings from the ACORN project will include publication in relevant national and international peer-reviewed journals as well as practitioner-friendly webpage dissemination.

The ACORN project has three initial core phases - all covered by the initial period of funding - that will be implemented over the first three years of the project (with a view to establishing a longer-term, programmatic initiative to be maintained over a much longer period). *Phase One* is focused upon the design, preparation and promotion activities. *Phase Two* involves distribution of a brief (2 page) baseline practitioner questionnaire, practitioner recruitment for the wider project (establishing a national chiropractor database), the launch of an Expression of Interest (EOI) round and initial approval for nested sub-studies and PhD proposals. Finally, *Phase Three* will facilitate additional and ongoing nested sub-study activity available to senior researchers and their teams and PhD development opportunities alongside maintaining the national chiropractor database. The ACORN project is available to those already developing and conducting chiropractic research to take advantage of a national chiropractic network to address a broad spectrum of related research questions.

In the remaining sections of this paper we outline the three phases of the ACORN project providing a general overview of the project’s approach and methods and highlighting how the project incorporates key design features which ensure short term and long term critical investigation and evaluation as well as the capacity building necessary to enable the profession and field to develop and prosper.

#### Phase one: ethics, design, preparation and promotion

In this initial phase of ACORN, the project Steering Committee is focused upon a number of platform-building tasks. It is imperative for a national-scale PBRN project such as ACORN to attract as high a response rate as possible [30] from the target population (*all* practising chiropractors across Australia) and critical to this is a well-developed promotion campaign. In response, Phase One of the project includes an extensive promotions strategy to be administered via regional and national events in order to reach and engage as many of the registered practising chiropractors across Australia as possible. To this end, ACORN senior Chief Investigators will present introductory ACORN project talks at relevant venues and events (including regional and national Executive and Membership Meetings, AGMs and research-focused events) and provide regular updates and information briefings via relevant newsletters (e.g. via Chiropractor’s Association of Australia and Chiropractic and Osteopathic College of Australasia) throughout 2014 and early 2015. Further information regarding the ACORN project will also be available via the project webpage [34].

Drawing upon previous relevant research in Australia and overseas [26,27] the ACORN project team will design, pilot and finalise a brief 2 page questionnaire which will be administered in Phase Two to collect baseline practitioner and practice data regarding chiropractor characteristics, practice characteristics, patient management and case-load mix amongst other topics. All the initial phase design features of the ACORN project will also be subject to ethical review and approval by relevant University Ethics Committees.

#### Phase two: administering a baseline chiropractor survey, establishing a national chiropractor research database and launching an expression of interest (EOI) call

An initial focus of Phase Two of the project will be to administer the questionnaire instrument designed and finalised in Phase One. The questionnaire will be as short as possible (maximum of 2 pages) to encourage and maximise a good response rate. Initial pilot-testing shows the questionnaire will take approximately 6–8 minutes to complete. The questionnaire will include examination of practitioner’s demographics, their practice characteristics including the frequency of patient types and presentations as well as the techniques and methods of care employed. The survey will be administered to *all* registered chiropractors across Australia (via both electronic and hard copy mail out) and will constitute the first national-scale mapping of chiropractors’ clinical management and patient care undertaken in Australia. In addition, the national mapping of chiropractic provided from the brief questionnaire will also be critical to establishing a broad data resource with which others can build, value-add, examine specific lines of investigation and address additional research questions.

Alongside the 2 page questionnaire, chiropractors will also receive an additional invitation to participate in the longer-term ACORN project. The contact details of each consenting chiropractor will be added to a secure database (to be securely housed at ARCCIM, UTS) which will be an ongoing resource for further research investigation via approved EOI applications (see below). The ACORN project has been assessed for formal learning hours and the allocation of Continuing Professional Development hours is subject to an individual’s level and amount of engagement.

Phase Two will also include an initial call for EOIs to be conducted by other interested researchers, practitioners and research teams/groups who may wish to access the ACORN chiropractor database for the purpose of developing and conducting nested sub-studies. Such EOIs regarding sub-study proposals will follow a formal application process and will be peer-reviewed by the ACORN Steering Committee headed by Senior ARCCIM academics with a view to ensuring appropriate standards of scientific validity, feasibility and design/methodological expertise are maintained.

Alongside accommodating more senior researchers, sub-study applications which primarily build upon or contain PhD studentships/study are also strongly encouraged. Indeed, all established chiropractic departments and research groups are encouraged to seek additional funding to facilitate separate PhD studentships/stipends which can be nested in the ACORN project and thereby take advantage of the infrastructure established by the project. Sub-studies suitable for EOI submission can include a vast array of possible designs and features including (but not restricted to): qualitative, quantitative and mixed-method research design; data collected via both chiropractors and/or their patients; and cross-sectional as well as longitudinal studies.

#### Phase three: facilitating chiropractor and patient Sub-studies, PhD development and further funding opportunities

In Phase Three the EOI call will remain open to encourage ongoing sub-study development and applications. The ACORN project database provides an ideal platform for nesting future PhD projects and can therefore be harnessed and utilised as a rich and valuable resource for doctoral level studies that will ultimately build chiropractic research capacity. In addition, the core ACORN project research team at ARCCIM will prepare further funding applications and seek input from relevant and interested partners to support applications for Federal Government research funding where possible. With this in mind, the ARCCIM will also explore opportunities to maintain and regularly update the ACORN practitioner database providing a valuable and ongoing resource for research and research capacity building in chiropractic over the three initial years of funding and beyond.

### Addressing key needs for research and research capacity building via the ACORN project

The ACORN project has been designed to directly address the key features essential to growing the broad evidence base and research capacity of Australian chiropractic (outlined earlier). These features are now outlined in more detail below.

#### Adopting a critical, non-partisan research approach built upon rigorous health and medical research methods and designs

Chiropractic, like other areas of health care practice located beyond conventional medicine, has generated emotive debate and commentary over recent years [35,36]. Such debate has tended to be a barrier to the scientific, rigorous examination and assessment of chiropractic within the Australian health care system. Indeed, there is an urgent need for chiropractic to be subject to critical investigation utilising rigorous designs and methods in order to promote a balanced, non-partisan approach to empirical study and appraisal – a situation no different to that demanded of all health care professions regardless of origins or orientation [37]. Such a balanced, non-partisan approach is at the very core of the ACORN project which has been designed and is independently led and conducted by a team of senior multi-disciplinary health care researchers (research methodologists) and yet inclusive of field-practitioner participation and input to assist in making the project more relevant to frontline practice. The ARCCIM team have extensive training and experience in successfully applying robust designs and methods to the critical study of health care topics (across complementary medicine, conventional health care and beyond) as well as established track records in attracting a significant portfolio of National Health and Medical Research Council (NHMRC) and Australian Research Council (ARC) grant and Fellowship funding.

#### Accommodating a coordinated, broad, multidisciplinary research focus

It is important that chiropractic research, at this significant juncture in its development, be co-ordinated and sufficiently broad (both disciplinarily and methodologically) in its focus as to ensure a suitable platform to accommodate as many sub-studies as possible. While increasing expertise and detailed engagement with specific areas of chiropractic care is wanting, the immediate task facing this research field is guaranteeing the research activity of today provides the most efficient use of resources and energy for future endeavours [38]. A PBRN design such as that adopted in the ACORN project is an excellent vehicle to ensure this is achieved within the real-life setting of frontline practice [39].

At the heart of the ACORN project is a multi-disciplinary approach incorporating a wide spectrum of clinical, public health and health services research methods, designs and disciplinary-based perspectives. The ACORN project (especially Phases Two and Three via the EOI process) encourages the conduct of a multitude of nested-study designs which can include observational studies and experimental studies.

#### First national scale, longitudinal project of its type in Australia

It is vital that any serious attempt to build a significant chiropractic research agenda and culture that will have substantial influence and impact within and beyond the profession be designed around a study of national scale. ACORN has been designed to provide the first national research project of its type in Australian chiropractic. Through recruitment from registered chiropractors across Australia, ACORN will be able to provide the first national-scale mapping of chiropractors’ clinical management and patient care undertaken in Australia. This scope of study also provides excellent opportunity for analyses to be large scale (national) as well as regionally and locally significant – the national data collected can also be analysed in terms of States/Territories, regional areas (i.e. a major urban area) or based upon urban/rural/remote settings amongst other criteria.

Similarly, PBRN designs such as that adopted by the ACORN project allow excellent infrastructure to conduct longitudinal analyses whereby patients and treatment outcomes are tracked over specific time periods. Such work is particularly important in addressing questions around the effectiveness of different treatments and will be strongly encouraged in terms of partnering and/or outside teams of investigators looking to submit sub-study proposals.

#### Sustainable and research capacity building

Essential to harnessing and promoting a well-developed research culture within chiropractic is a commitment to ensuring research activity is more than short-term and involves developing the research training and careers of those within chiropractic. Chiropractic, not dissimilar to other professions outside of conventional health care, is in much need of research growth starting with expanding the pool of people with PhDs in the field [38]. In response, the ACORN project actively encourages and comfortably accommodates PhD candidates and other early career researchers via the EOI process. Crucially, the ACORN project also facilitates not only immediate but also mid to long-term research activity around chiropractic in much needed areas - the ACORN project effectively establishes the necessary infrastructure upon which others can build and value-add.

The EOI and nested sub-study design also provide excellent opportunity for chiropractors to engage in conducting empirical research study. As previous PBRN studies have illustrated, so long as time and effort is taken to promote closer researcher-practitioner relations and develop research collaborations where practitioners are mentored by senior researchers, a PBRN such as the ACORN project can comfortably accommodate those chiropractors looking to develop their research experience and skills [30].

#### Incorporating practitioner engagement and reflective of practice realities

A PBRN design such as that adopted within the ACORN project necessitates that the research perspective be directly linked to and contextualised within everyday practice activity and behaviours. The ACORN project design – drawing upon data from both grass-roots practitioners and their patients – will ensure that the research focus is of direct relevance to (and thereby impact upon) practice realities. As with other quality health research, the ACORN project will accommodate an ability to approach the practitioner and/or patient as *a partner/partners* in investigations and the project acknowledges the essential engagement and involvement of both chiropractors and their patients in ensuring the project and project findings are translational and impactful for patient care. The ACORN project provides a national platform for chiropractors in the field to directly participate within significant research projects, which can influence the future of the profession in Australia.

#### Encouraging inclusivity and research network building

In order to maximise and help support the pockets of research excellence and activity already established around chiropractic, the ACORN project houses a fundamentally *inclusive* research design. The success of the project is significantly dependent upon the engagement and commitment of interested researchers and research teams (which can, and in many cases should, include key practitioner collaborators) beyond the core ARCCIM investigators. Indeed, the EOI process has been developed to encourage partnership and input beyond the immediate ARCCIM team and the ACORN project actively encourages the submission of nested sub-studies led by qualified and interested groups/parties across Australia and/or on the international stage.

## Conclusion

It is imperative that chiropractors and chiropractic profession, like all health care practitioners and practices currently available and utilised by Australians, be subject to sufficient critical, rigorous examination and assessment. While addressing the many research gaps on this topic via a coordinated effort has obvious and far-reaching benefits for chiropractors and the wider profession in Australia, the ACORN project also constitutes a significant initiative for others beyond the profession. To examine and assess fundamental aspects and features of contemporary chiropractic will produce insights of benefit to all interested and engaged in researching, practicing and managing evidence-based, safe and effective patient care.

Addressing the research and research capacity building needs of Australian chiropractic will certainly require long-term commitment and engagement from a number of stakeholders across the profession (especially practitioners and researchers). However, the establishment of the ACORN project constitutes a solid national platform for helping address these needs in an inclusive manner which ensures investigations can accommodate an appropriate wealth of interests, designs and expertise as well as provide an evidence-base assessment and reflect the grass-roots features of chiropractic patient care.

## References

[1] Xue CC, Zhang AL, Vin C, Myers R, Polus B, Story DF (2008). Acupuncture, chiropractic and osteopathy use in Australia: a national population survey. BMC Public Health.

[2] Adams J, Sibbritt D, Broom A, Loxton D, Pirotta M, Humphreys J (2011). A comparison of complementary and alternative medicine users and use across geographical areas: a national survey of 1,427 women. BMC Complement Altern Med.

[3] Adams J, Sibbritt D, Lui C (2011). The urban–rural divide in complementary and alternative medicine use: a longitudinal study of 10,638 women. BMC Complement Altern Med.

[4] French SD, Densley K, Charity MJ, Gunn JM (2013). Who uses chiropractic services?. Chiropr Man Therap.

[5] Sibbritt D, Adams J (2010). Back pain amongst 8,910 young Australian women: a longitudinal analysis of the use of conventional providers, complementary and alternative medicine (CAM) practitioners and self-prescribed CAM. Clin Rheumtol.

[6] Steel A, Adams J, Sibbritt D, Broom A, Gallois C, Frawley J (2012). Utilisation of complementary and alternative medicine (CAM) practitioners within maternity care provision: results from a nationally representative cohort study of 1,835 pregnant women. BMC Pregnancy Childbirth.

[7] Wardle J, Sibbritt D, Adams J (2013). Referrals to chiropractors and osteopaths: a survey of general practitioners in rural and regional New South Wales, Australia. Chiropr Man Therap.

[8] Xue C, Zhang AL, Lin V, Da Costa C, Story DF (2007). Complementary and alternative medicine use in Australia: a national population-based survey. J Altern Complement Med.

[9] Zodet M, Stevans J (2012). The 2008 prevalence of chiropractic use in the US adult population. J Manipulative Physiol Ther.

[10] Brown BT, Bonello R, Fernandez-Caamano R, Eaton S, Graham PL, Green H (2014). Consumer characteristics and perceptions of chiropractic and chiropractic services in Australia: results from a cross-sectional survey. J Manipulative Physiol Ther.

[11] Rubenstein SM, Bolton J, Webb AC, Hartvigsen J (2014). The first research agenda for the chiropractic profession in Europe. Chiropr Man Therap.

[12] Mootz RD, Coulter ID, Hansen DT (1996). Health services research related to chiropractic: review and recommendations for research prioritization by the chiropractic profession. J Manipulative Physiol Ther.

[13] Triano JJ, Goertz C, Weeks J, Murphy DR, Kranz KC, McClelland GC (2006). Chiropractic in North America: toward a strategic plan for professional renewal-outcomes from the 2006 chiropractic strategic planning conference. J Manipulative Physiol Ther.

[14] Adams J, Broom A, Jennaway M (2008). Qualitative methods in chiropractic research: one framework for future enquiry. J Manipulative Physiol Ther.

[15] Stuber K, Bussieres A, Gotlib A (2009). Chiropractic research capacity in Canada in 2008. J Can Chiropr Assoc.

[16] Adams J, Sibbritt D, Broom A, Wardle J, Steel A, Murthy V, Adams J, Andrews G, Barnes J, Broom A, Magin P (2012). Research capacity building in traditional, complementary and integrative medicine: grass-roots action towards a broader vision. Traditional, complementary and integrative medicine: an international reader 2012.

[17] Farmer E, Weston K (2002). A conceptual model for capacity building in Australian primary health care research. Aust Fam Physician.

[18] Hay AD, Rortviet G, Purdy S, Adams J, Sanci LA, Schermer TR (2012). Primary care research – an international responsibility. Fam Pract.

[19] Van Weel C, Rosser WW (2004). Improving health care globally: a critical review of the necessity of family medicine research and recommendations to build research capacity. Ann Fam Med.

[20] Adams J, Smith A (2003). Qualitative methods in radiography research: a proposed framework. Radiography.

[21] Britt HC, Miller GC (2000). The BEACH study of general practice. Med J Aust.

[22] Green LA, Dovey SM (2001). Practice based primary care research networks: they work and are ready for full development and support. Br Med J.

[23] Green LA, Hickner J (2006). A short history of primary care practice-based research networks: from concept to essential research laboratories. J Am Board Fam Med.

[24] MacLaughlin EJ, Ardery G, Jackson E, Ives T, Young R, Fike D (2013). Institutional review board barriers and solutions encountered in the collaboration among pharmacists and physicians to improve outcomes now study: a national multicentre practice-based implementation trial. Pharmacother.

[25] Gilbert GH, Williams OD, Rindal DB, Pihlstrom DJ, Benjamin PL, Wallace MC (2008). The creation and development of the dental practice-based research network. J Am Dent Assoc.

[26] Hawk C, Long CR, Boulanger KT (2001). Prevalence of nonmusculoskeletal complaints in chiropractic practice: report from a practice-based research program. J Manipulative Physiol Ther.

[27] French SD, Charity ML, Forsdike K, Gunn JM, Polus BI, Walker BF (2013). Chiropractic observation and analysis study (COAST): providing an understanding of current chiropractic practice. Med J Aust.

[28] Jones C (2006). Laboratories of primary care practice-based networks in Canada. Can Fam Physician.

[29] Dwan KM, Magin PJ (2008). The desire for research in general practice. Aust Fam Physician.

[30] Gilbert GH, Richman JS, Gordan VV, Rindal DB, Fellows JL, Benjamin PL (2011). Lessons learned during the conduct of clinical studies in the dental PBRN. J Dent Educ.

[31] Pearce KA, Love MM, Barron MA, Matheny SC, Mahfoud ZM (2004). How and why to study the practice content of a practice-based research network. Ann Fam Med.

[32] Andrews J, Pearce K, Love M (2005). Information-seeking behaviours of practitioners in a primary care practice-based research network (PBRN). J Med Libr Assoc.

[33] Curro F, Vena D, Naftolin F, Terracio L, Thompson V (2012). The PBRN initiative: transforming new technologies to improve patient care. J Dent Res.

[34] ACORN Project webpage. Available at http://www.acorn-arccim.com/. 2015. Accessed 30 January 2015.

[35] Dwyer J. There’s no place for pseudo-scientific chiropractic in Australian universities. The Conversation 6 Dec 2011. Available at http://theconversation.com/theres-no-place-for-pseudo-scientific-chiropractic-in-australian-universities-4576. 2011. Accessed 30 January 2015.

[36] O’Neill A, Willis E (1994). Chiropractic and the politics of health care. Aust N Z J Public Health.

[37] Adams J (2008). Utilising and promoting public health and health services research in complementary and alternative medicine: the founding of NORPHCAM. Complement Ther Med.

[38] Swain M, Downie A, Brown B, Lystad RP (2013). A commentary to address the state of chiropractic research in Australia. Chiropr J Aust.

[39] Peterson KA, Lipman PD, Lange CJ, Cohen RA, Durako S (2012). Supporting better science in primary care: a description of practice-based research networks (PBRNs) in 2011. J Am Board Fam Med.

